# Integration of retinal layer thinning into NEDA-3 predicts disability progression in multiple sclerosis

**DOI:** 10.1007/s00415-026-13909-8

**Published:** 2026-06-09

**Authors:** Fabian Föttinger, Nik Krajnc, Klaus Berek, Jan Philipp Nolte, Martin Heinrich Schmidauer, Franziska Di Pauli, Barbara Kornek, Fritz Leutmezer, Stefan Macher, Tobias Monschein, Markus Ponleitner, Paulus Rommer, Christiane Schmied, Karin Zebenholzer, Gudrun Zulehner, Tobias Zrzavy, Florian Deisenhammer, Thomas Berger, Berthold Pemp, Harald Hegen, Gabriel Bsteh

**Affiliations:** 1 Department of Neurology, Medical University of Vienna, Waehringer Guertel 18-20, 1090 Vienna, Austria; 2 Comprehensive Center for Clinical Neurosciences and Mental Health, Medical University of Vienna, Vienna, Austria; 3 Department of Neurology, Medical University of Innsbruck, Anichstraße 35, 6020 Innsbruck, Austria; 4 Department of Ophthalmology, Medical University of Vienna, Vienna, Austria

**Keywords:** Multiple sclerosis, Optical coherence tomography, Disability, Progression, No evidence of disease activity

## Abstract

**Introduction:**

Retinal layer thinning is associated with disability progression and treatment failure in relapsing multiple sclerosis (RMS). However, the systematic integration of optical coherence tomography (OCT)-derived metrics into a composite measure of treatment response has not yet been evaluated.

**Methods:**

We analyzed two observational cohorts of patients with RMS who newly initiated DMT, received an MRI and OCT at baseline and 12 months, and had ≥ 24 months of clinical follow-up. No evidence of disease activity (NEDA-3/NEDA-3 + OCT) status was assigned at 12 months after DMT. Retinal thinning was defined as a reduction of ≥ 1.0 µm/year peripapillary retinal nerve fiber layer or ≥ 0.5 µm/year ganglion-cell/inner plexiform layer. The primary endpoint was confirmed disability progression occurring after the 12 month NEDA assessment. Both low-efficacy DMT and high-efficacy were included and analyzed jointly, with treatment class entered as a covariate in all models.

**Results:**

Overall, 124 individuals (72% female, mean age 33.1 [SD ± 7.7] years, median EDSS of 2.0 [IQR 0.0–2.5]) were included. Over a median follow-up period of 3.4 years, disability progression was observed in 28 (23%) individuals.

Time to and risk of disability progression did not significantly differ between EDA-3 and NEDA-3 (restricted mean survival time [RMST]: 43.4 [SE ± 2.8] vs. 48.1 [SE ± 1.3] months, p = 0.067; adjusted hazard ratio [aHR] 1.52, 95% LL-CI 0.64, p = 0.173). When retinal layer thinning was incorporated, EDA-3 + OCT was associated with a higher risk of future progression (aHR 6.59, 95% LL-CI 2.38, p = 0.005) and shorter time to progression (RMST: 41.9 [SE ± 2.1] vs. 51.9 [SE ± 0.9] months, p < 0.001).

**Conclusion:**

Incorporating retinal layer thinning into the NEDA-3 framework substantially improves prediction of subsequent disability progression compared with conventional NEDA-3 alone, identifying a subgroup of patients in whom ongoing neurodegeneration appears to drive disability accumulation despite suppressed inflammatory activity.

**Supplementary Information:**

The online version contains supplementary material available at 10.1007/s00415-026-13909-8.

## Introduction

Over the past three decades a multitude of disease-modifying treatments (DMT) has been established for the management of relapsing multiple sclerosis (RMS) [1]. While the treatment paradigm is shifting towards early suppression of inflammatory disease activity, the definition of this treatment target remains debated. Amongst several proposed composite measures, the most prominent concept is “no evidence of disease activity-3” (NEDA-3), defined as absence of relapse, disability progression, and MRI activity. NEDA-3 is now widely used both in trials and in clinical practice to benchmark treatment success [2]. However, NEDA-3 is insensitive to ongoing subclinical neuroaxonal damage, particularly under high-efficacy DMT (HE-DMT), where overt inflammatory activity is often efficiently suppressed [3]. In parallel, neurodegenerative processes are a major driver of disease progression in RMS, even in early stages [4, 5]. Recognizing that diffuse neurodegeneration drives long-term disability, several groups have proposed extending NEDA by adding structural biomarkers of tissue loss [2, 6, 7].

Optical coherence tomography (OCT) provides a paraclinical surrogate marker to quantify the extent and persistence of neuroaxonal loss by measuring inner retinal layer thickness through individual and repeated measurements. OCT predicts future disability progression in RMS and could provide a viable approach to a refined measure of treatment response [8–11]. However, OCT has not yet been integrated systematically into the NEDA-3 composite as a marker of treatment response.

We therefore aimed to investigate whether integration of a retinal thinning threshold into conventional NEDA-3 (NEDA-3 + OCT) after one year of treatment would improve prediction of disability progression in a prospective bicentric cohort.

## Methods

### Study design and participants

**Fig. 1:**
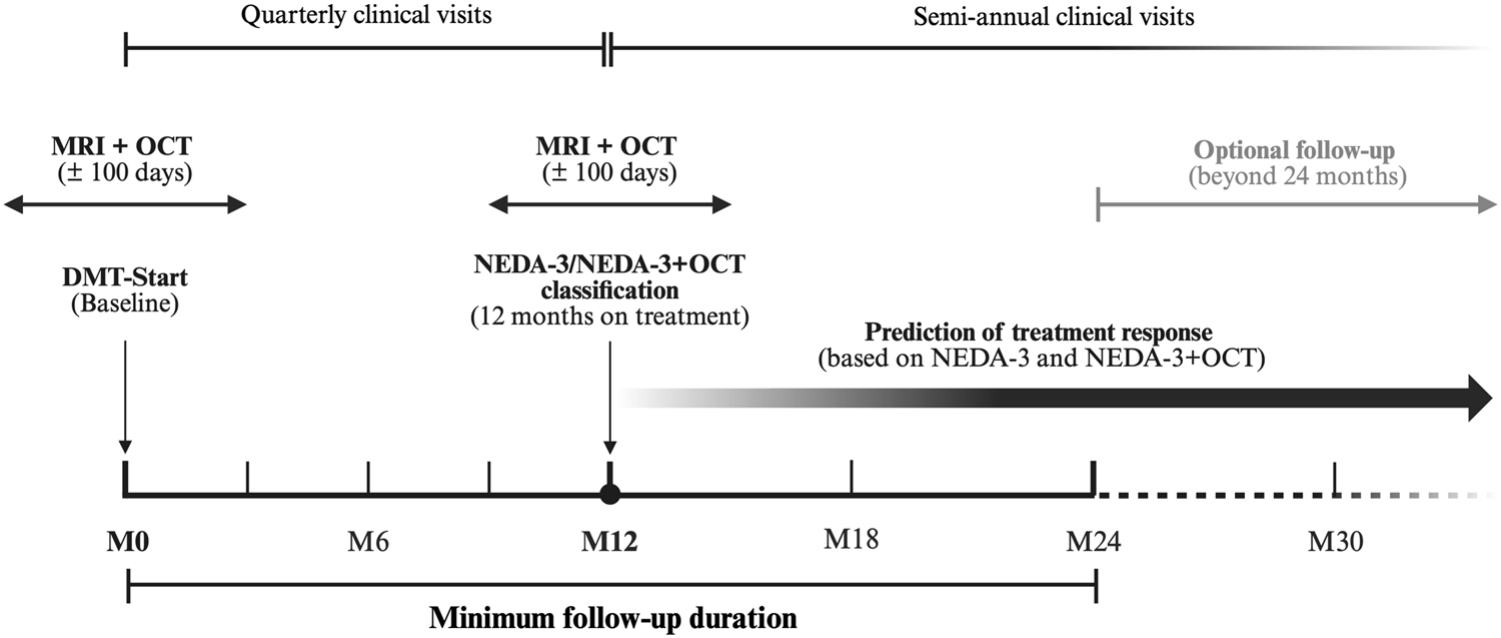
Study design. Eligibility for analysis of prospectively collected data required ≥ 24 months of documented follow-up upon initiation of DMT, with quarterly visits during the first treatment year and semiannual visits thereafter. MRI and OCT were obtained at baseline and at 12 months. NEDA-3 and NEDA-3 + OCT status was assessed 12 months after DMT initiation, and its utility for predicting subsequent disability progression was evaluated. *DMT* disease-modifying treatment, *OCT*   optical coherence tomography, *MRI*   magnetic resonance imaging; NEDA-3/EDA-3 = “No Evidence of Disease Activity – 3”/ “Evidence of Disease Activity – 3”; NEDA-3 + OCT/ EDA-3 + OCT = expanded NEDA-3/EDA-3 definition

**Fig. 2:**
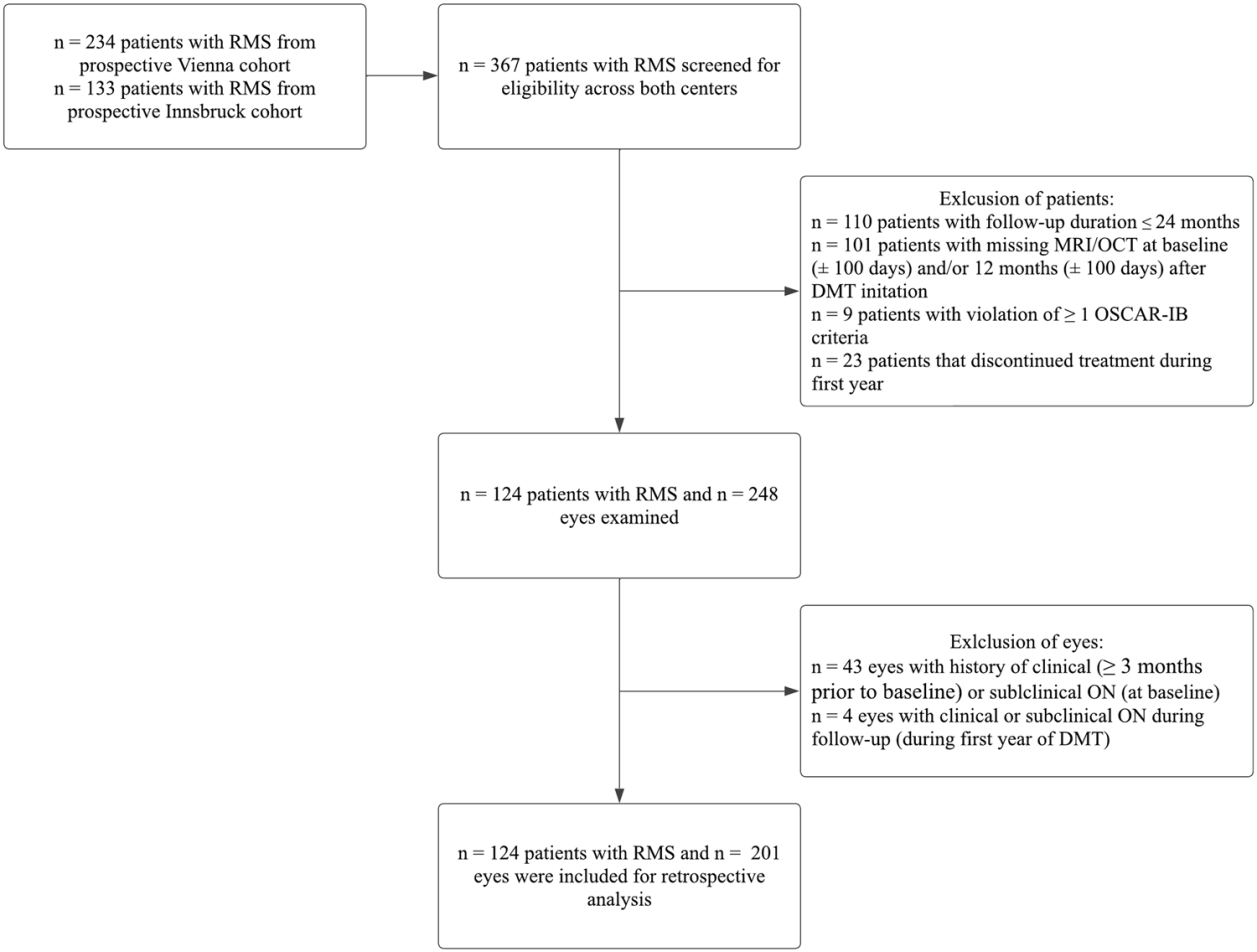
Patient selection flowchart. Depicts the sequential application of inclusion and exclusion criteria that yielded the final cohort. OSCAR-IB criteria according to Tewarie P. et al., 2012 [14]. *RMS*   relapsing multiple sclerosis, *MRI*  magnetic resonance imaging, *OCT*   optical coherence tomography, *ON*   optic neuritis, *DMT*   disease-modifying treatment

For this study, we screened patients from two ongoing prospective observational cohort studies conducted at the Departments of Neurology of the Medical Universities of Vienna and Innsbruck. Both cohorts prospectively enroll patients with RMS diagnosed according to 2017 McDonald criteria initiating a DMT. [12] Patients were eligible for inclusion if: i) the DMT was maintained for at least 12 months, and ii) a documented clinical follow-up of at least 24 months was available. Magnetic resonance imaging (MRI) and OCT were mandatory at treatment initiation (± 100 days) and at 12 months (± 100 days) for inclusion in the current analysis. EDSS assessments were performed during routine clinical visits and examiners were blinded to OCT findings and derived NEDA classifications. The study design and detailed inclusion/exclusion process are shown in Figs. 1 and 2. For this analysis, the database was locked on July 1st 2025.

### Standard protocol approvals, registrations, and patient consents

The study was approved by the ethics committee of the Medical University Vienna (ethical approval number: 1378/2020) and the Medical University of Innsbruck (ethical approval number: AM3743-281/4.3). Written informed consent was obtained from all study participants. This study adheres to the reporting guidelines outlined within the “Strengthening the Reporting of Observational Studies in Epidemiology” (STROBE) statement.

### Examinations

#### Magnetic resonance imaging

Brain MRI was performed on 3 T scanners using harmonized protocols comprising T2-weighted, 3D fluid-attenuated inversion recovery and pre-/post-contrast T1-weighted sequences. New T2 lesions (T2L) and contrast-enhancing lesions (CEL) 12 months after starting DMT relative to baseline were quantified by experienced neuroradiologists or neurologists at each site in accordance with standardized recommendations.

#### Optical coherence tomography

OCT imaging was performed by experienced neuro-ophthalmologists on spectral-domain OCT (Spectralis^®^, Heidelberg Engineering, Heidelberg, Germany; software Heidelberg Eye Explorer). For the assessment of average peripapillary retinal nerve fiber layer (pRNFL) thickness, a 12° (3.4 mm) circular scan (1536 A-scans, automatic real-time tracking (ART) averaging 100 frames), centered on the optic nerve head, was recorded. To assess ganglion-cell and inner plexiform layer (GCIPL) thickness, macular imaging was performed by applying a 20 × 20° volume scan centered on the fovea (25 vertically aligned B-scans, 512 A-scans, ART: 16 frames). The follow-up function was activated to ensure that repeated measurements were obtained from the same retinal locations. GCIPL thickness was calculated as the mean value of the inner (3 mm ring) and outer (6 mm ring) macular quadrants as defined by the circular Early Treatment Diabetic Retinopathy Study (ETDRS) grid [13]. Semiautomated segmentation was performed through the integrated manufacturer’s software and manual correction of obvious delineation errors. All imaging conformed to the OSCAR-IB quality control standards. [14]

As for the definition of the OCT criterion integrated into NEDA-3 + OCT, baseline and follow-up measurements were compared and a reduction at 12 months in the GCIPL thickness of ≥ 0.5 µm/year and/or a reduction in the pRNFL thickness of ≥ 1.0 µm/year were defined as a clinically significant change exceeding the 95th percentile in healthy controls [11, 15]. By default, thicknesses of GCIPL and pRNFL were calculated as the binocular mean, unless a prior episode of optic neuritis (ON) or an interocular asymmetry suggestive of subclinical ON was detected, defined as a difference of ≥ 4 µm for GCIPL and/or ≥ 5 µm for pRNFL, in which case the less-affected eye alone was analyzed [16]. Eyes that developed ON between baseline and 12 months were excluded and only the values of eyes without ON and subclinical ON during the observation period were used for calculation of retinal layer thinning. All longitudinal OCT progression events were confirmed in a follow-up examination ≥ 6 months after the 12 month OCT examination to ensure sustained retinal layer thinning.

The study’s methodology was reported in accordance with the revised Advised Protocol for OCT Study Terminology and Elements (APOSTEL 2.0) recommendations. [17]

### Endpoints and definitions

The primary endpoint was defined as confirmed disability progression (defined as an increase in the “Expanded Disability Status Scale” [EDSS] of ≥ 1.5/1.0/0.5 points from a roving baseline score of 0/1.0–5.5/ ≥ 6.0, persistent for ≥ 6 months) occurring at ≥ 12 months after DMT initiation.

The secondary endpoint was defined as disability accrual, that is a composite outcome consisting of disability progression as defined above and/or any of the following, each confirmed after ≥ 6 months: (i) a ≥ 20% prolongation in the Timed 25-Foot Walk (T25FW) and/or (ii) a ≥ 20% prolongation in the 9-Hole Peg Test (9HPT) and/or (iii) a ≥ 10% (or ≥ 4-point) reduction in the Symbol Digit Modalities Test (SDMT).

Tertiary endpoints were disability progression and disability accrual independent of relapse activity (PIRA-EDSS and PIRA disability accrual, respectively), i.e. disability progression and disability accrual as defined above not preceded by relapse since the previous assessment, remaining above the progression threshold throughout a confirmation of 6 months where only scores not preceded by relapse within 30 days are used for confirmation. [18]

Secondary endpoints and tertiary endpoints were predefined as exploratory analyses included for the purpose of sensitivity testing and completeness.

NEDA-3 was defined as the absence of relapses, disability progression (as defined above), and MRI activity (≥ 2 new T2 lesions and/or ≥ 1 gadolinium-enhancing lesion) at 12 months after treatment initiation. [19]

The extended NEDA-3 + OCT definition incorporated retinal layer thinning criteria measured by quantifying longitudinal change of the pRNFL and/or GCIPL thickness. Not fulfilling the respective ≥ 1 of the NEDA-3/NEDA-3 + OCT criterion constituted evidence of disease activity (EDA), classified as EDA-3 or EDA-3 + OCT, respectively.

DMTs were grouped in either low-efficacy DMT (LE-DMT), including interferon-beta (IFNb), glatiramer acetate (GLAT), teriflunomide (TER), dimethyl fumarate (DMF), or HE-DMT, including sphingosine-1-phosphate receptor modulators (S1PM; fingolimod [FTY], ozanimod [OZA], siponimod [SIP] or ponesimod [PON]), antiCD20-monoclonal antibodies (ofatumumab [OFA], ocrelizumab [OCR] or rituximab [RTX]), natalizumab (NTZ) or alemtuzumab (ATZ).

### Statistical analysis

Statistical analysis was performed in R (version 4.4.0) [20] using the survival [21] and pROC [22] packages. Categorical variables are summarized as absolute frequencies and percentages, whereas continuous measurements are reported either as mean and standard deviation (SD) or median and inter-quartile range (IQR; 25th—75th percentiles), as appropriate. Group-wise univariate comparisons of variables were assessed with Pearson’s χ^2^ test or Mann–Whitney U test, as appropriate.

Time-to-event functions for the primary endpoint (6 month confirmed disability progression) as well as for the secondary and tertiary endpoints (disability accrual, PIRA-EDSS, PIRA disability accrual) stratified by attainment of NEDA-3 (versus EDA-3) and NEDA-3 + OCT (versus EDA-3 + OCT), respectively, were visualized with Kaplan-Meier curves and adjusted survival curves derived from multivariable Cox models, as detailed below. Treatment switches occurring after the 12 month NEDA assessment were retained in the primary analysis and classified according to the baseline DMT category. This approach was chosen because the primary prognostic question addressed the extent to which 12 month NEDA-3/NEDA-3 + OCT status predicts subsequent disability progression, and the analysis was therefore conducted in an intention-to-treat–like design. Nevertheless, to evaluate the robustness of our primary analysis, treatment switching was additionally addressed in two sensitivity analyses by (i) modelling treatment switch as a time-varying covariate, and (ii) by censoring follow-up at the time of treatment switch. Global comparisons across strata defined by evidence of disease activity were performed with the log-rank test and restricted mean survival time (RMST) with corresponding standard errors (SE) being reported.

Determinants of time to event were quantified in multivariable Cox proportional-hazards models. The independent variable was failure to attain NEDA-3 or NEDA-3 + OCT status after one year of ongoing treatment. Potential effect modifications were assessed by adding an interaction term between baseline treatment class (LE-DMT or HE-DMT) and NEDA-3/NEDA-3 + OCT status in a separate Cox model. Sub-group-specific hazard ratios were estimated for each treatment class. To mitigate confounding, an individual propensity score (PS) including sex, age, disease duration, baseline EDSS, relapse count in the year before baseline (continuous variable), MRI T2-lesion load (number of T2-hyperintense lesions) and treatment class (binary: LE-DMT or HE-DMT) was estimated by logistic regression. The PS was entered as an additional covariate in all Cox models. All effect estimates of Cox regression analysis are presented as adjusted hazard ratios (aHR) with one-sided 95% confidence intervals (CI). Potential multicollinearity was evaluated with variance-inflation factors (VIF), and the proportional-hazards assumption was checked with scaled Schoenfeld residuals. Model fit was assessed via Akaike Information Criterion (AIC) and likelihood ratio tests. Model discrimination was quantified using the concordance index (Harrell’s c-index).

Sensitivity analyses explored alternative definitions of the OCT criterion including GCIPL-only and pRNFL-only retinal thinning, as well as different threshold definitions for retinal layer thinning. We furthermore expressed retinal atrophy change as age-normalized longitudinal *z*-scores drawn from an external reference population [23]. The z-score difference between baseline and month 12 (Δz = z_Month-12_ – z_Baseline_) was then dichotomized at the optimal cut-point identified by receiver-operating-characteristic (ROC) analysis of the primary endpoint, using Youden-index. As an additional sensitivity analysis, OCT progression within the NEDA-3 + OCT construct was redefined using 12 month thinning thresholds relative to baseline thicknesses (≥ 1.0%/year pRNFL and/or ≥ 0.5%/year GCIPL) to assess robustness of the primary findings to flooring effect. To address potential differences arising from alternative MRI activity definitions, a sensitivity analysis applying a more proactive threshold of ≥ 1 new T2 lesion was performed.

Based on our pre-specified one-sided hypotheses, one-sided hypothesis testing was used. Prior studies have provided consistent evidence on the direction of the effects of the independent variables (used in the Cox regression models) on the dependent variables (primary and secondary endpoints). Specifically, higher MRI activity (greater number of T2-hyperintense lesions and/or presence of CEL), [24, 25] higher relapse rate, [26] higher age, [27] disease duration, [28] higher baseline EDSS, [29] and use of LE-DMT versus HE-DMT [30–32] have all been associated with an increased risk of disability progression in RMS. In the context of OCT, multiple studies have provided sufficient evidence that increased pRNFL and/or GCIPL thinning is associated with higher risk of subsequent disability accumulation. [8, 9, 23, 33] Given the absence of plausible rationale for an opposite effect, statistical testing for the primary and secondary endpoints was performed using one-sided p values and one-sided 95% CI, reporting either the lower limit (LL) or upper limit (UL) as appropriate. P-values < 0.05 were considered statistically significant.

## Results

Of 367 patients with RMS included in the ongoing prospective cohort studies at database closure, 124 patients (mean age 33.1 [SD ± 7.7] years, female 89/124 [72%]) met the inclusion criteria for the present analysis (Fig. 2). Table 1 summarizes characteristics of the study cohort. Fifty-eight patients (47%) were initiated on a LE-DMT and 66 (53%) on HE-DMT.

**Table 1:** Patient characteristics of included patients. Individuals are grouped according to NEDA-3/EDA-3 status and NEDA-3 + OCT/EDA-3 + OCT status after one year of treatment

		N/EDA-3 (1 year)			N/EDA-3 + OCT (1 year)		
Variable	Overall N = 124	NEDA-3 N = 91	EDA-3 N = 33	p Value	NEDA-3 + OCT N = 67	EDA-3 + OCT N = 57	p Value
Sex^1^				0.059^†^			0.01^†^
Male	35 (28)	21 (23)	14 (42)		12 (18)	23 (40)	
Female	89 (72)	70 (77)	19 (58)		55 (82)	34 (60)	
Age (y)^3^	33.1 (7.7)	34.2 (7.6)	29.9 (7.3)	0.005^‡^	34.2 (7.5)	31.8 (7.8)	0.351^‡^
Disease duration (y)^3^	3.4 (4.8)	4.2 (5.2)	1.1 (1.7)	< 0.001^‡^	4.5 (5.3)	2.1 (3.6)	< 0.001^‡^
Number of treatments prior to Baseline^1^				0.1^†^*			< 0.001^†^*
None	70 (56)	47 (52)	23 (70)		27 (40)	43 (75)	
1	32 (26)	24 (26)	8 (24)		22 (33)	10 (18)	
2	17 (14)	16 (18)	1 (3)		14 (21)	3 (5)	
3	3 (2)	2 (2)	1 (3)		2 (3)	1 (2)	
4	3 (2)	2 (2)	0		2 (3)	0 (0)	
Relapse 1 year prior to DMT^1^				0.239^†^			0.166^†^
No	30 (24)	25 (27)	5 (15)		20 (30)	10 (18)	
Yes	94 (76)	66 (73)	28 (85)		40 (70)	54 (82)	
Baseline DMT^1^				0.098^†^			0.045^†^
LE-DMT *IFNß* *GLAT* *DMF* *TERI*	58 (47) *3 (2)* *10 (8)* *37 (30)* *8 (7)*	38 (42) *2 (2)* *6 (7)* *26 (29)* *4 (4)*	20 (61) *1 (3)* *4 (12)* *11 (34)* *4 (12)*		24 (36) *2 (3)* *4 (6)* *18 (27)* *0 (0)*	34 (60) *1 (2)* *6 (11)* *19 (33)* *8 (14)*	
HE-DMT *S1P* *CLAD* *AntiCD20* *NAT*	66 (53*)* *18 (14)* *23 (19)* *18 (14)* *7 (6)*	53 (58) *14 (15)* *17 (19)* *17 (19)* *5 (5)*	13 (39*)* *4 (12)* *6 (18)* *1 (3)* *2 (6)*		43 (64) *8 (12)* *15 (22)* *16 (24)* *4 (6)*	23 (40) *10 (18)* *8 (14)* *2 (3)* *3 (5)*	
MRI T2-Lesions^2^	10 (7, 15)	10 (7, 16)	11 (8, 13)	0.723^‡^	9.0 (7, 17)	10 (7.0, 14)	0.853^‡^
EDSS Baseline^2^	2 (0, 2.5)	2 (0, 2.5)	2 (0, 2.0)	0.403^‡^	2 (0, 2.5)	2 (0, 2)	0.538^‡^
EDSS at Last Follow-up^2^	2 (1, 3)	2 (0, 3)	2.5 (2, 3)	0.086^‡^	2 (0, 2.5)	2.5 (2.0, 3.0)	< 0.001^‡^

^1^Frequency (%), ^2^Median (IQR), ^3^Mean (SD)

^†^χ^2^ test, ^‡^Mann–Whitney *U* test

*No treatment prior to baseline compared to ≥ 1 treatment prior to baseline

*NEDA-3/ EDA-3*   “no evidence of disease activity – 3”/ “evidence of disease activity – 3”, *NEDA-3 + OCT/ EDA-3 + OCT* expanded NEDA-3/ EDA-3 definition, *OCT*   optical coherence tomography, *DMT*   disease-modifying treatment, *LE-DMT* low-efficacy DMT, *HE-DMT*   high-efficacy DMT, *IFNß*   interferon-beta, *GLAT*  glatiramer acetate, *DMF*   dimethyl fumarate, *TERI *  teriflunomide, *S1P*   sphingosin-1 phosphate receptor modulator, *CLAD*   cladribine, *AntiCD20*   anti-CD20 monoclonal antibody, *NAT*  natalizumab, *MRI*  magnetic resonance imaging, *EDSS*  expanded disability status scale

During a median follow-up of 3.5 years (IQR 2.7 – 4.1), disability progression was reported in 28 individuals (23%), of which 17 (61%) represented PIRA events. Disability progression during follow-up occurred numerically more frequently among participants classified as EDA-3 at one year compared to those achieving NEDA-3 (30% [10/33] versus 20% [18/91]; p = 0.216). Using the NEDA-3 + OCT classification, the corresponding rates for disability progression during follow-up were 40% (23/57) for EDA-3 + OCT versus 18% (12/67) for NEDA-3 + OCT. Of the patients classified as EDA-3 after 12 months, 15 (45%) lost NEDA-3 status due to relapse, 15 (45%) due to isolated MRI activity and only three (10%) due to disability progression. Among the 91 individuals who fulfilled NEDA-3 criteria at 12 months, 25 (28%) individuals demonstrated retinal layer thinning during the first year of DMT. Applying NEDA-3 + OCT definition consequently reduced the proportion of patients classified as free of disease activity from 73% (NEDA-3) to 54% (NEDA-3 + OCT), with 57 (46%) individuals categorized as EDA-3 + OCT. OCT worsening at 12 months was associated with an overall trend to more disability progression events, both in NEDA-3 and EDA-3 groups (Suppl. Figure S1).

### NEDA-3 vs. NEDA-3 + OCT in predicting risk of disease progression

**Fig. 3:**
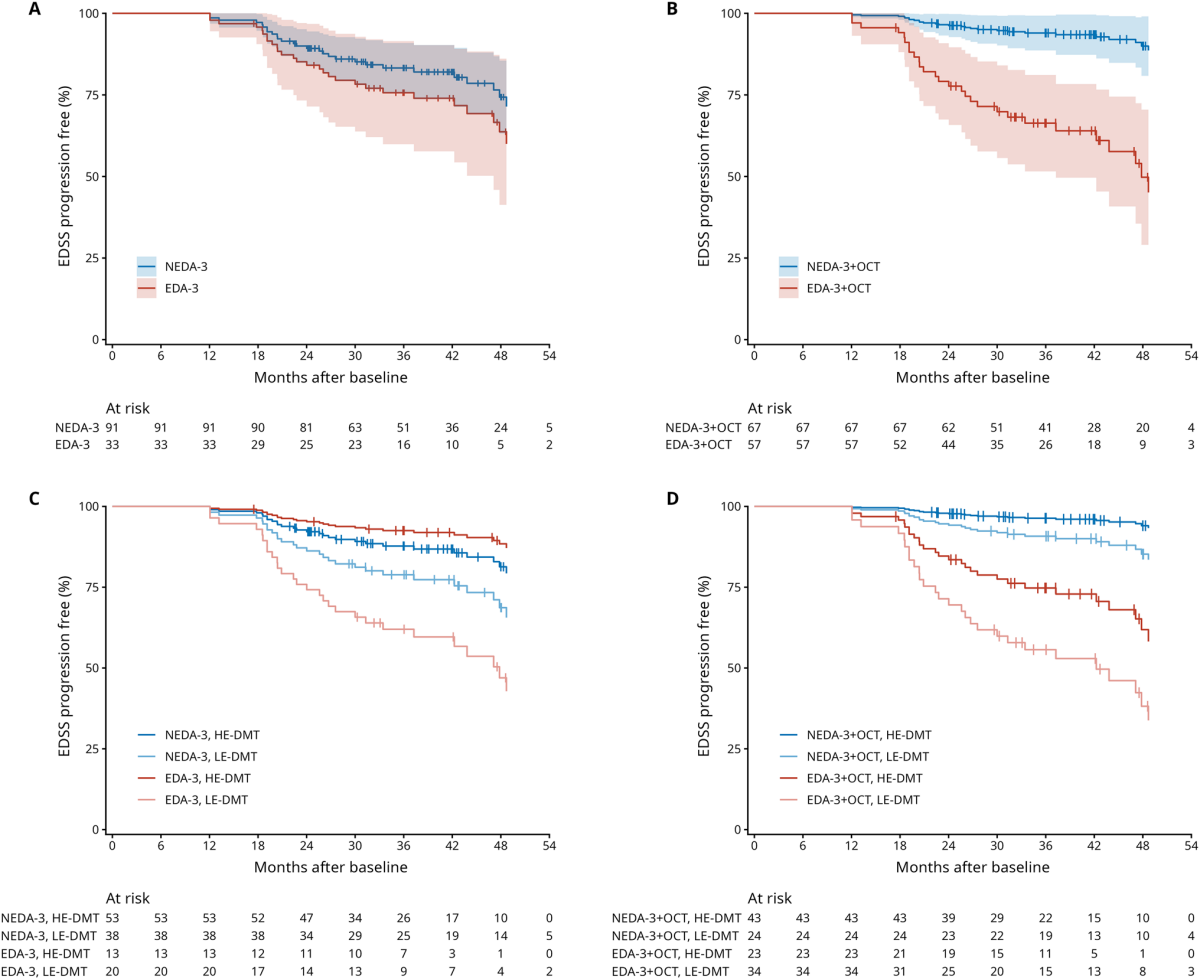
Adjusted survival curve according to NEDA status and primary endpoint (disability progression). In panel (**A**) and (**B**) 95%-CI are depicted. In panel (**C**) and (**D**) patients are stratified according to DMT class at baseline. Adjusted survival curves are derived from multivariable Cox proportional-hazards models adjusted for age, sex, disease duration, baseline EDSS, relapse count in the year prior to baseline, baseline MRI T2-lesion count, and baseline DMT category (low- vs. high-efficacy). *CI* confidence interval, NEDA-3/EDA-3 = “No Evidence of Disease Activity – 3″/”Evidence of Disease Activity – 3″; NEDA-3 + OCT/EDA-3 + OCT = expanded NEDA-3/EDA-3 definition; *DMT*  disease-modifying treatment, *LE-DMT*   low-efficacy DMT, *HE-DMT*   high-efficacy DMT

In survival analysis, conventional one year NEDA-3 status was associated with a longer difference in cumulative disability progression-free survival during follow-up (RMST months for NEDA-3 48.1 [SE ± 1.3] versus EDA-3 43.4 [SE ± 2.8], log-rank p = 0.067, Fig. 3A**, **Suppl. Figure S2A), although this difference did not meet statistical significance. In contrast, NEDA-3 + OCT status produced a markedly greater stratification, with a significant separation of the survival curves (RMST in months for NEDA-3 + OCT 51.9 [SE ± 0.9] versus EDA-3 + OCT 41.2 [SE ± 2.1], log-rank p < 0.001; Fig. 3B**, **Suppl. Figure S2B).

In the PS-adjusted Cox regression model, NEDA-3 status conferred a directionally consistent, yet marginal increase in risk for future disability progression failing to meet statistical significance (adjusted hazard ratio [aHR] 1.52, 95% LL-CI 0.64, p = 0.173). Conversely, failure to achieve NEDA-3 + OCT conferred an aHR of 6.59 for future disability progression (95% LL-CI 2.38, p = 0.005). Applying NEDA-3 + OCT status improved model discrimination in parallel (concordance of 0.716 ± 0.043 for NEDA-3 + OCT vs. concordance of 0.607 ± 0.059 for NEDA-3) and produced overall better goodness of fit (AIC = 236.89 vs. AIC = 249.31 and likelihood-ratio test χ^2^ = 20.02, p < 0.001 for NEDA-3 + OCT versus χ^2^ = 2.7, p = 0.3 for NEDA-3). Retinal layer-specific analyses produced similar effect estimates, showing neither substantial attenuation nor amplification (Table 2). After accounting for treatment switching in sensitivity analyses, either by modelling treatment switch as a time-varying covariate or by censoring follow-up at the time of switching, the direction of the associations remained unchanged (Suppl. Table S2).

**Table 2:** Multivariable Cox Regression analyses for independent variables (EDA-3 or EDA-3 + OCT status at one year)

					95% CI	
	Coefficient	SE	aHR	p-Value	Lower limit	Upper limit
EDA-3	0.421	0.446	1.523	0.173	0.635	–
EDA-3 + OCT (pRNFL and/or GCIPL)	1.886	0.541	6.589	0.005	2.384	–
EDA-3 + OCT (pRNFL)	1.561	0.481	4.764	0.001	1.856	–
EDA-3 + OCT (GCIPL)	1.799	0.489	6.043	< 0.001	2.318	–
EDA-3 HE-DMT*	−0.476	0.650	0.594	0.317	–	3.59
EDA-3 + OCT HE-DMT*	2.1034	0.8521	8.194	0.014	1.542	–

Risk of disability progression grouped according to EDA classification

Adjustment of the models with age, sex, EDSS at baseline, disease duration, relapse ≤ 1 year prior to baseline, baseline MRI T2-lesion count and baseline DMT (binary: LE-DMT/HE-DMT) using a propensity-score adjusted approach

No multicollinearity was evident (all VIF < 1.7)

A p-value < 0.05 was considered statistically significant, one-sided 95% CI is shown

^*^Subgroup-specific contrast within the HE-DMT stratum from a Cox-regression model with an interaction term (patients receiving HE-DMT n = 66 [53%])

*aHR*  adjusted hazard ratio, *CI*  confidence interval, *NEDA-3/ EDA-3*   “no evidence of disease activity – 3”/ “evidence of disease activity – 3”, *NEDA-3 + OCT/ EDA-3 + OCT*  expanded NEDA-3/EDA-3 definition, *OCT*   optical coherence tomography, *pRNFL*   peripapillary retinal nerve fiber layer, *GCIPL*  ganglion cell and inner plexiform layer, *HE-DMT*  high efficacy disease-modifying therapy, *EDSS*  expanded disability status scale

In subgroup-specific analyses considering DMT class, EDA-3 + OCT status was significantly associated with an increased risk of disability progression in both patients on HE-DMT (aHR = 8.19, 95% LL-CI = 1.54, p = 0.014) as well as on LE-DMT (aHR = 5.29, 95% LL-CI = 1.55, p = 0.008, Fig. 3D). The overall model, which incorporated NEDA-3 + OCT status, baseline DMT category, and the respective interaction term, indicated a consistent effect of NEDA-3 + OCT status across treatment classes (p = 0.483). A comparable pattern was observed for classical NEDA-3 status in different DMT categories. EDA-3 showed a trend for higher risk of disability progression in the LE-DMT subgroup (aHR = 2.01, 95% LL-CI = 0.90, p = 0.077), while no such association was evident in the HE-DMT subgroup (aHR = 0.59, 95% UL-CI = 3.59, p = 0.317, Fig. 3C).

Across sensitivity analyses, including alternative pRNFL/GCIPL thresholds, relative pRNFL/GCIPL changes, one year changes in age-adjusted z-scores for both layers, and stratification by baseline layer thickness, the findings were directionally consistent with the primary results (Suppl. Table S3–S6; Suppl. Figure S3, S4). Additionally, applying the alternative MRI activity threshold of ≥ 1 new T2 lesion yielded directionally identical results, with no meaningful change in effect estimates for either NEDA-3 or NEDA-3 + OCT (data not shown).

### Secondary and tertiary endpoints – disability accrual and PIRA

Composite secondary and tertiary disability accrual endpoints occurred exclusively in participants who met EDA-3 + OCT criteria and consequently no events were observed in the NEDA-3 + OCT subgroup, precluding the use of Cox regression. However, log-rank test confirmed a marked group difference for all disability accrual endpoints (log-rank p < 0.001 for all disability accrual endpoints; Suppl. Figure S6A, B).

For PIRA-EDSS, 18 events were reported overall with log-rank statistics indicating a significant discrimination of PIRA-EDSS events when grouped according to NEDA-3 + OCT status (p = 0.002, Suppl. Figure S6C). Cox-regression analysis indicated that EDA-3 + OCT was a significant predictor of PIRA-EDSS (aHR = 8.84, 95% LL-CI 2.23, p = 0.002, Suppl. Table S7). Sensitivity analysis with age-adjusted z-scores yielded comparable discrimination across all tertiary endpoints (Suppl. Table S5).

## Discussion

This study aimed to investigate whether integration of retinal layer atrophy, an established marker of neuroaxonal loss in MS, into NEDA-3 during the first year of DMT treatment improves the prediction of treatment response measured by future disability progression.

Using a multivariable approach in a prospectively collected multicenter dataset, we demonstrate that incorporation of retinal layer (pRNFL and GCIPL) thinning (“NEDA-3 + OCT”) after one year of ongoing treatment markedly improves prediction of short- to mid-term treatment response compared to conventional NEDA-3/EDA-3 status. While the conventional NEDA-3 composite displayed a consistent, but modest, directional association with subsequent disability worsening, OCT reclassified one fourth of the study population deemed “stable” by NEDA-3 into the EDA-3 + OCT category, almost halving the proportion considered to be free of disease activity from 73 to 54%. Attainment of EDA-3 + OCT, independent of the retinal layer used (GCIPL, pRNFL or both), conferred a more than sixfold higher risk of future disability progression, driven largely by PIRA events (61%). Notably, the impact of retinal thinning was more pronounced in individuals with HE-DMT as in those treated with LE-DMT, indicating that OCT may identify a phenotype at risk for disability progression, where focal inflammatory activity is suppressed and ongoing neurodegeneration may emerge as the predominant driver of subsequent disability progression.

In the era of HE-DMT with the aim to efficiently suppress inflammatory disease activity, there is an unmet need for more refined measures of treatment target beyond clinical and radiological indicators of inflammation. Our findings underline that NEDA-3 criteria capture clinically meaningful focal inflammatory treatment effects, but have limited sensitivity for detecting disability progression driven by ongoing neurodegeneration and, thus, only insufficiently delineate true treatment response [3, 7, 34, 35]. In our cohort the difference in disability progression between NEDA-3 and EDA-3 categories was most apparent among individuals with stable retinal layer thickness, while patients with retinal thinning showed comparably high progression rates regardless of NEDA-3 status (Suppl. Figure S1). This pattern suggests that, in the absence of pronounced neurodegeneration, conventional NEDA-3 remains a useful marker of treatment response, whereas in patients with widespread CNS neuroaxonal loss, neurodegenerative processes emerge as the dominant determinant of disability progression and may attenuate the predictive value of a focal inflammation-centred NEDA-3 construct. Additionally, prior long-term studies report that among patients who attained NEDA-3 at 2 years on initial treatment, approximately 26% still went on to develop disability worsening [35]. The pooled OPERA I and II trial analyses suggested that 78–89% of disability accumulation was PIRA in early RMS even on HE-DMT, a pattern reflected in the high frequency of PIRA events in our cohort (61%), implying necessity for more sensitive monitoring measures. [5]

As such, OCT has emerged as a fast, non-invasive, readily available and cost-effective tool to quantify neuroaxonal damage in MS by measuring thinning of pRNFL and GCIPL, which in particular reflect widespread axonal loss and also correlates with cortical and deep grey-matter atrophy [36–38]. While cross-sectional studies have demonstrated that OCT carries prognostic value in predicting future disability progression, the concept of incorporating longitudinal OCT changes in monitoring disease activity to better understand disease trajectories has only been emphasized recently [8, 39, 40]. The effect sizes we observed for EDA-3 + OCT status align with those reported in a recent meta-analysis of longitudinal OCT measures as prognostic biomarkers in MS [9]. Additionally, prior evidence on treatment-related retinal layer atrophy further supports OCT as a biomarker of therapeutic response, as recently emphasized by meta‑analysis of 2158 individuals which suggest that HE‑DMT attenuates GCIPL thinning significantly more than LE-DMT. [10, 11, 40]

Alternative approaches to address this gap have been investigated as well. Brain-volume loss (BVL) gained prominence, as several studies have reported that early or accelerated brain atrophy predicts subsequent disability accumulation in RRMS [41–43]. Adding BVL to NEDA-3 using a 0.4%/year BVL threshold (“NEDA-4”) in patients treated with fingolimod reduced the proportion of patients fulfilling NEDA from 31.0% to 19.7% on fingolimod, a similar effect size as observed in the current analysis [6]. A systematic review of 11 cohorts (1846 participants) confirmed that meeting NEDA-4 at 2 years doubled the odds of remaining progression-free [34]. However, routine assessment of BVL is limited by availability due to high technical demands and methodological concerns such as pseudoatrophy and reliability issues due to field strength, sequence and post-processing, to which OCT seems to be more resilient [44]. Test–retest studies using OCT demonstrate excellent reliability of pRNFL and GCIPL thickness measures (with coefficients of variation on the order of 1% and intraclass correlation > 0.98 in experienced centers), enabling confident detection of true change over time, provided stringent quality control is applied [45, 46]. As such OCT may be the more practical tool for early detection of treatment failure due to its sensitivity, feasibility and relative robustness in the short term.

The findings of this study have several key implications, as OCT refines stratification for risk of future disability worsening, significant retinal layer thinning may provide additional information to guide early escalation from LE-DMT to HE-DMT. From a clinical perspective, patients treated with HE-DMT that exhibit significant retinal layer thinning, although currently faced with limited further treatment options, may benefit from novel treatments targeting compartmentalized inflammation and/or neurodegeneration.

From a research perspective, OCT may provide a promising treatment marker of neurodegeneration in clinical trials targeting neurodegenerative pathways and may be worth studying in terms of guiding early treatment stratification.

The strength of this work lies in the quality and uniformity of its data set. All participants were drawn from two Austrian tertiary MS centers that prospectively collect clinical, MRI and OCT data under harmonized protocols. OCT images passed OSCAR-IB quality filters and MRI was acquired with a standardized sequence set, minimizing measurement noise across sites.

Nevertheless, several limitations warrant consideration. First, the sample size of the study and consequently the overall number of disability progression events was modest, particularly in the HE-DMT stratum, wherefore our findings require external validation in independent cohorts. That said, concordant hazard-ratio estimates, large effect sizes, and a non-significant treatment-class interaction support, however, the validity of the OCT signal. While data were obtained from prospective observational cohorts, the endpoints and eligibility parameters applied in this analysis were determined retrospectively, introducing the potential for selection bias. Of the initial cohort (n = 396), 31.3% fulfilled the inclusion requirements (see Fig. 1), yet no substantial differences were observed in the demographic baseline variables when comparing the complete cohort with the current study population (Supplementary Figure S1). Furthermore, baseline disability levels in our cohort were comparatively low (median EDSS 2.0 [IQR 0.0–2.5]), reflecting an early-disease, predominantly mildly disabled population. Although absolute changes in EDSS between groups were therefore modest in magnitude, this likely reflects the early disease stage rather than a diminished biological signal.

Brain atrophy parameters could not be included in the analysis precluding a direct comparison between NEDA-3 + OCT composite and NEDA-4 (BVL) construct [47]. However, retinal layer thinning was consistently shown to strongly correlate with whole-brain atrophy, suggesting that the lack of MRI-based BVL is unlikely to diminish the robustness and validity of the current analysis. [36, 37]

All OCT scans were obtained on the Heidelberg Spectralis platform with a predefined acquisition protocol and strict quality control measures were applied to OCT scans with confounding factors rigorously excluded (e.g., severe myopia, ophthalmological damage unrelated to MS). Although inter-device reliability is good after rigorous quality control, variability in protocols and segmentation software could temper generalizability, which is why our findings are not directly applicable to other OCT devices [14, 45, 46]. Additionally, prior studies suggest that early post-treatment retinal thinning may partly reflect pre-treatment disease activity, and that this artifact could be mitigated by re-baselining, a factor we could not account for. However, we do not anticipate inflation of the effect-size estimates (aHRs). Instead, re-baselining would be expected primarily to enhance precision and narrow the confidence intervals [40]. Furthermore, to mitigate potential misclassification due to the absence of a formal MRI re-baselining window, we applied a more stringent MRI activity threshold of ≥ 2 new T2 lesions and/or ≥ 1 contrast-enhancing lesion, thereby reducing the risk of overestimating loss of NEDA-3.

Lastly, although our operational definition of OCT progression (≥ 1 µm/year pRNFL or ≥ 0.5 µm/year GCIPL loss over 12 months) may, in some individuals, approximate per-eye test–retest variability (≈1 µm for pRNFL and 0.5 µm for GCIPL, respectively), we selected absolute change thresholds in combination with inter-eye averaging and confirmation at a follow-up examination performed ≥ 6 months after the 12 month scan for several reasons. In particular, absolute change measures are generally more feasibly interpretable at the individual-patient level, whereas percentage-based thresholds may be better suited for group-level analyses because they normalize to baseline retinal layer thickness and are less susceptible to floor-effect bias, particularly in eyes with advanced pre-existing atrophy. In line with this, sensitivity analyses based on percentage change yielded directionally similar results. Although some degree of individual-level misclassification cannot be excluded, the findings remained robust across multiple sensitivity analyses, including stricter absolute thresholds (≥ 2 µm/year pRNFL and ≥ 1 µm/year GCIPL), relative cut-offs (≥ 1.0%/year pRNFL and/or ≥ 0.5%/year GCIPL over 12 months), and age-adjusted z-score criteria, all of which produced similar effect estimates. The selected absolute cut-offs are also supported by external data indicating that annual thinning rates > 0.5 µm/year for GCIPL and > 1.0 µm/year for pRNFL exceed the 95th percentile of retinal layer thinning in healthy controls [15]. Nonetheless, future studies should refine individualized thresholds and incorporate multi-visit confirmation strategies to further optimize the balance between sensitivity and specificity.

## Conclusion

Integrating longitudinal OCT-derived retinal atrophy into the NEDA frameework refines treatment monitoring in RMS by capturing neuroaxonal loss that persists beneath inflammatory quiescence. While conventional NEDA-3 retains predictive utility primarily in patients with overt inflammatory activity, the addition of OCT identifies an otherwise unrecognizable subgroup in whom neurodegeneration appears to be the principal driver of subsequent disability accumulation. Given its accessibility, OCT merits consideration as a complementary component of composite treatment targets alongside clinical and other radiological indices.

## Supplementary Information

Below is the link to the electronic supplementary material.

Supplementary file1 (DOCX 2371 KB)

Supplementary file2 (DOCX 31 KB)

## Data Availability

Anonymized data supporting the findings of this study are available from the corresponding author upon reasonable request by a qualified researcher and upon approval by the data-clearing committees of the Medical Universities of Vienna and Innsbruck.
