# Supplementary material for: Integration of retinal layer thinning into NEDA-3 predicts disability progression in multiple sclerosis
Source: J Neurol. 2026 Jun 9;273(7):382. doi: 10.1007/s00415-026-13909-8 (PMC13249778; doi:10.1007/s00415-026-13909-8)
Supplement: Supplementary file 1 — Supplementary file1 (DOCX 2371 KB) [file 415_2026_13909_MOESM1_ESM.docx]

**Supplementary Figures**

**
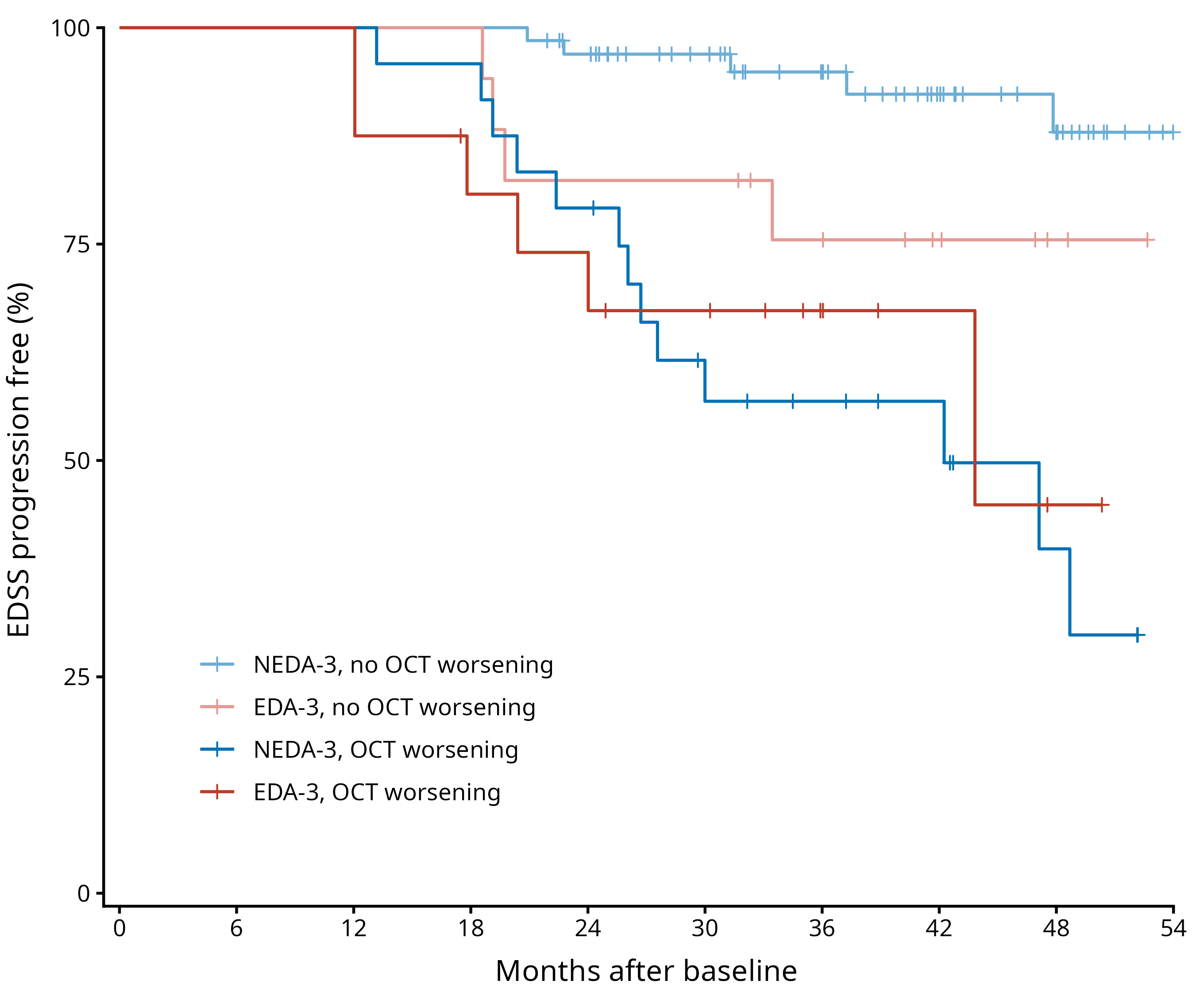
**

**Suppl. Figure S1.** **Kaplan-Meier curves grouped by NEDA-3 status and worsening in OCT at 12 months of treatment.** Definition of OCT worsening as reported in methods.

NEDA-3/EDA-3 = “No Evidence of Disease Activity – 3”/“Evidence of Disease Activity – 3”; OCT = Optical coherence tomography


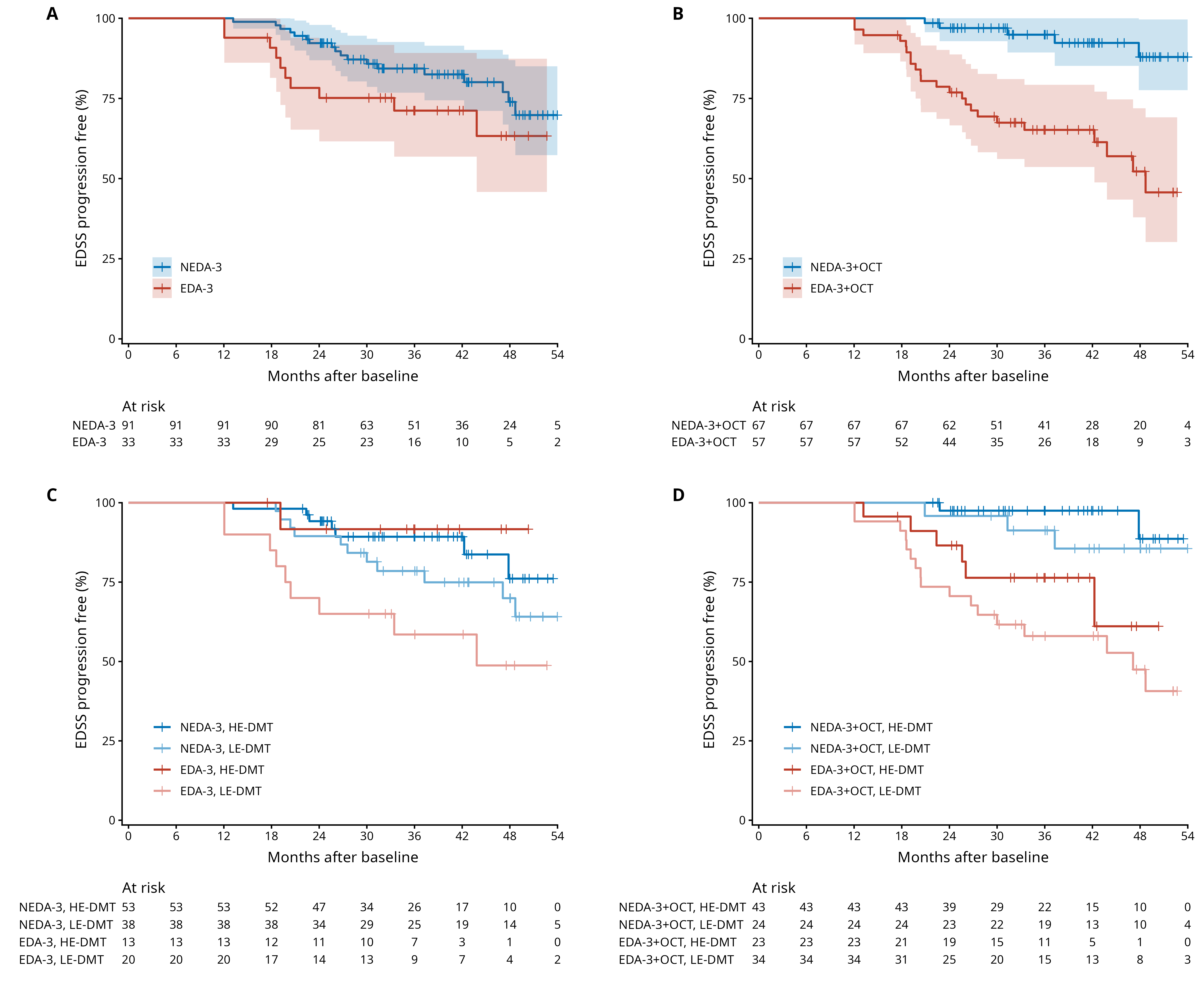


**Suppl. Figure S2. Kaplan-Meier curves according to NEDA status and primary endpoint (disability progression).** In panel (A) and (B) 95%-CI are depicted. In panel (C) and (D) patients are stratified according to DMT class at baseline.

CI = Confidence interval, NEDA-3/EDA-3 = “No Evidence of Disease Activity – 3”/”Evidence of Disease Activity – 3”; NEDA-3+OCT/EDA-3+OCT = expanded NEDA-3/EDA-3 definition; DMT = disease-modifying treatment; LE-DMT = low-efficacy DMT; HE-DMT = high-efficacy DMT


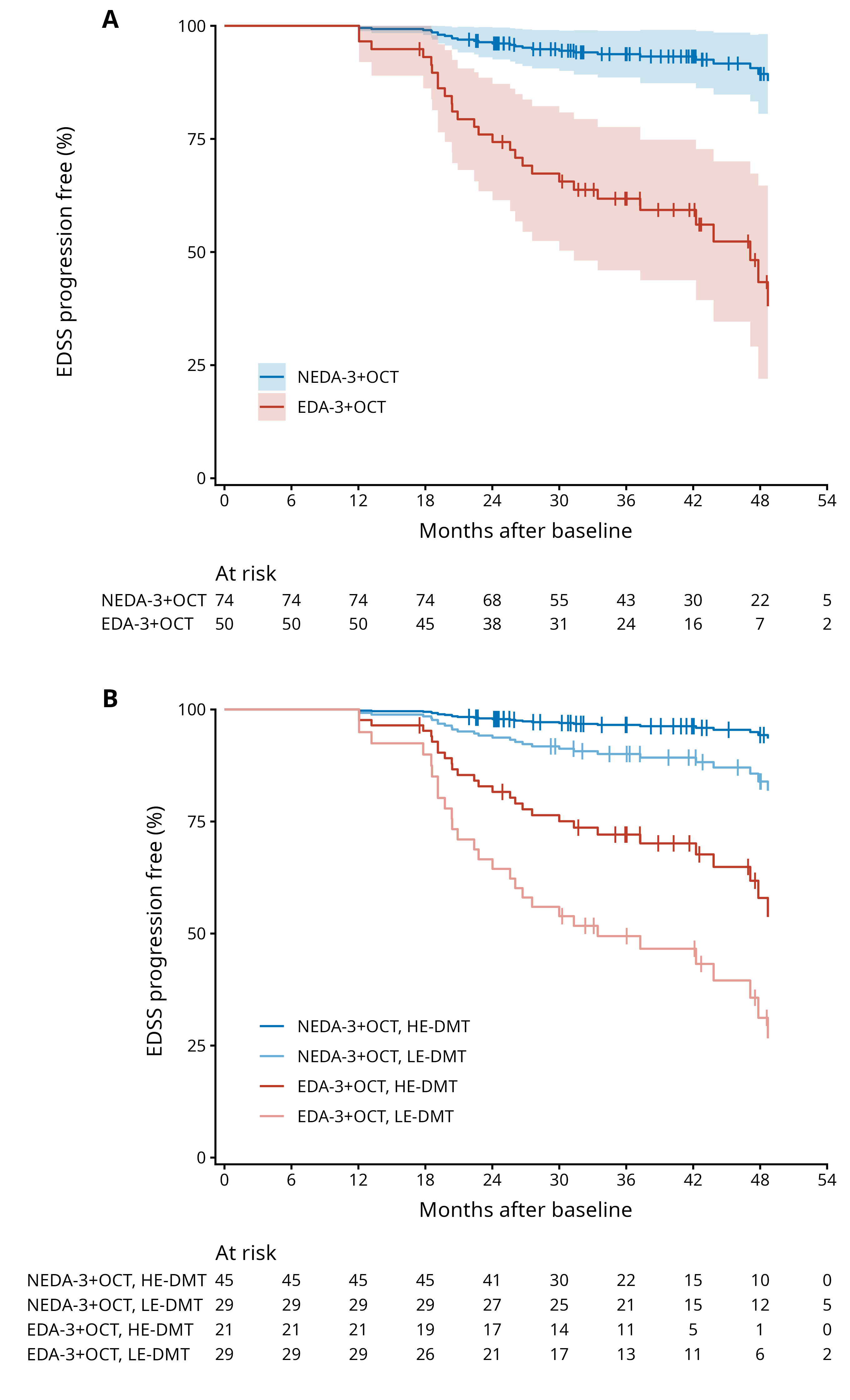


**Suppl. Figure S3.** **Adjusted survival curve according to NEDA-3+OCT status (with different layer thickness criterion) and disability progression.** Thresholds for significant reduction of retinal layer thickness at 12 months were defined as ≥ 1 µm/year for GCIPL and ≥ 2.0 µm/year for pRNFL.

Adjusted survival curves are derived from multivariable Cox proportional-hazards models adjusted for age, sex, disease duration, baseline EDSS, relapse count in the year prior to baseline, baseline MRI T2-lesion count, and baseline DMT category (low- vs. high-efficacy).

NEDA-3/EDA-3 = “No Evidence of Disease Activity – 3”/“Evidence of Disease Activity – 3”; NEDA-3+OCT/EDA-3+OCT = expanded NEDA-3/EDA-3 definition; OCT = Optical coherence tomography; MRI = Magnetic resonance imaging; LE-DMT = low-efficacy disease-modifying treatment; HE-DMT = high-efficacy disease-modifying treatment


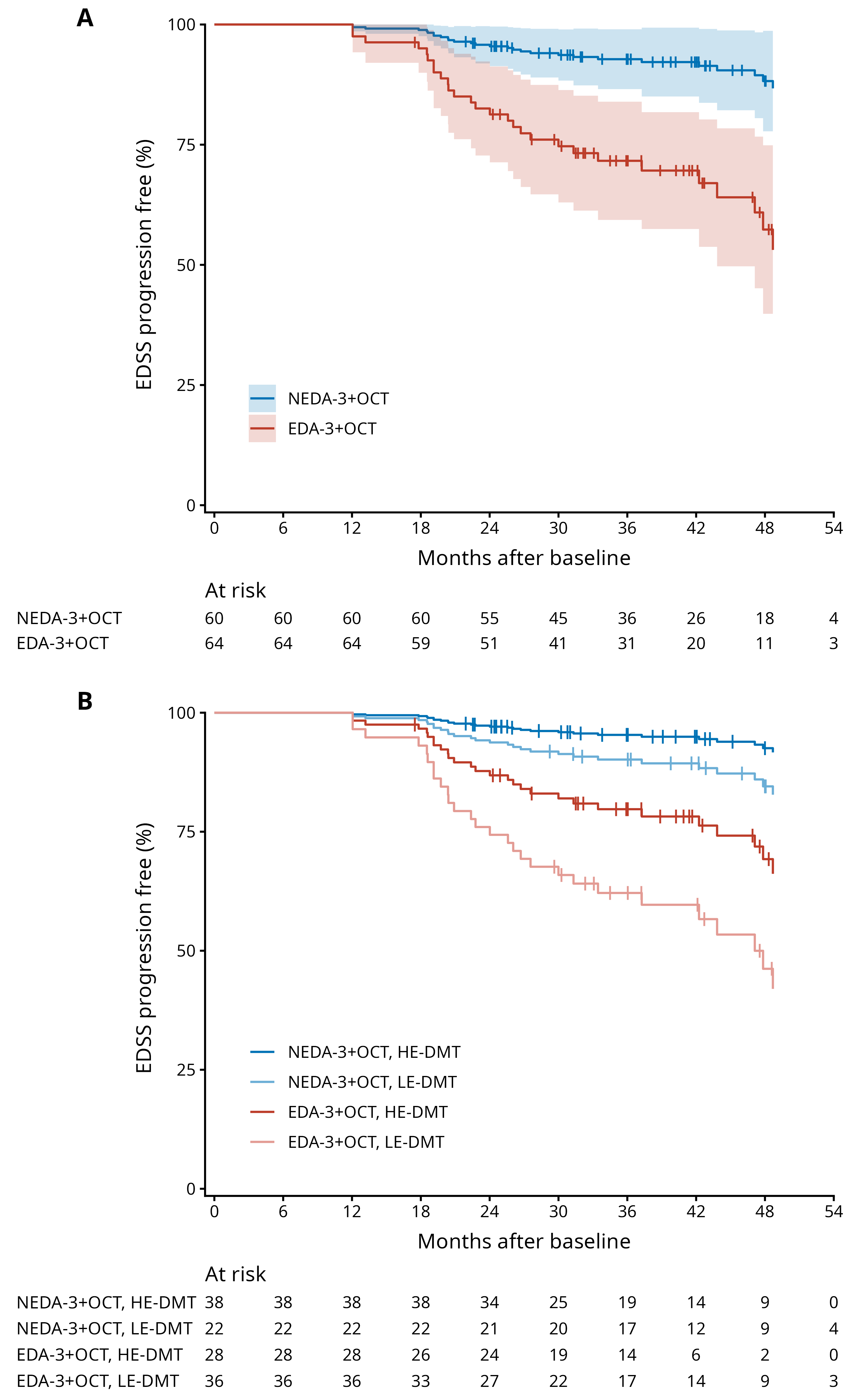


**Suppl. Figure S4.** **Adjusted survival curve according to NEDA-3+OCT status (with relative layer thickness criterion) and disability progression.** Thresholds for significant reduction of retinal layer thickness at 12 months were defined as ≥ 0.5%/year for GCIPL and ≥ 1.0%/year for pRNFL.

Adjusted survival curves are derived from multivariable Cox proportional-hazards models adjusted for age, sex, disease duration, baseline EDSS, relapse count in the year prior to baseline, baseline MRI T2-lesion count, and baseline DMT category (low- vs. high-efficacy).

NEDA-3/EDA-3 = “No Evidence of Disease Activity – 3”/“Evidence of Disease Activity – 3”; NEDA-3+OCT/EDA-3+OCT = expanded NEDA-3/EDA-3 definition; OCT = Optical coherence tomography; MRI = Magnetic resonance imaging; LE-DMT = low-efficacy disease-modifying treatment; HE-DMT = high-efficacy disease-modifying treatment.


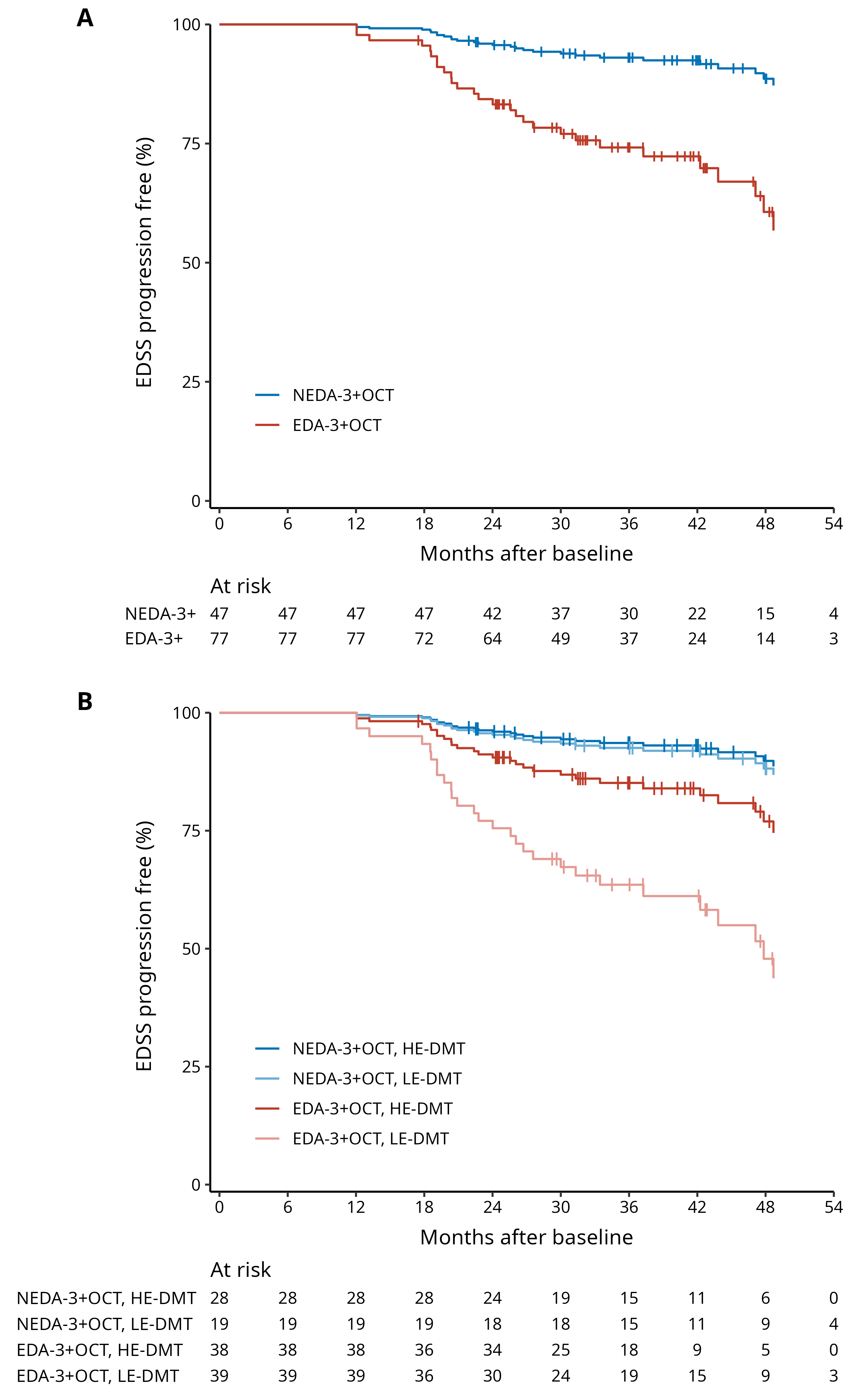


**Suppl. Figure S5. Adjusted survival curve according to NEDA status based on OCT ≈≈≈≈≈≈≈ and disability progression.** In panel (B) stratified according to DMT class at baseline.

Adjusted survival curves are derived from multivariable Cox proportional-hazards models adjusted for age, sex, disease duration, baseline EDSS, relapse count in the year prior to baseline, baseline MRI T2-lesion count, and baseline DMT category (low- vs. high-efficacy).

Receiver-operating-characteristic (ROC) analyses yielded optimal absolute *z*-score difference thresholds of 0.11 for pRNFL and 0.064 for GCIPL to discriminate NEDA-3+OCT/EDA-3+OCT status.

NEDA-3/EDA-3 = “No Evidence of Disease Activity – 3”/“Evidence of Disease Activity – 3”; NEDA-3+OCT/EDA-3+OCT = expanded NEDA-3/EDA-3 definition; OCT = Optical coherence tomography; MRI = Magnetic resonance imaging; LE-DMT = low-efficacy disease-modifying treatment; HE-DMT = high-efficacy disease-modifying treatment.

**
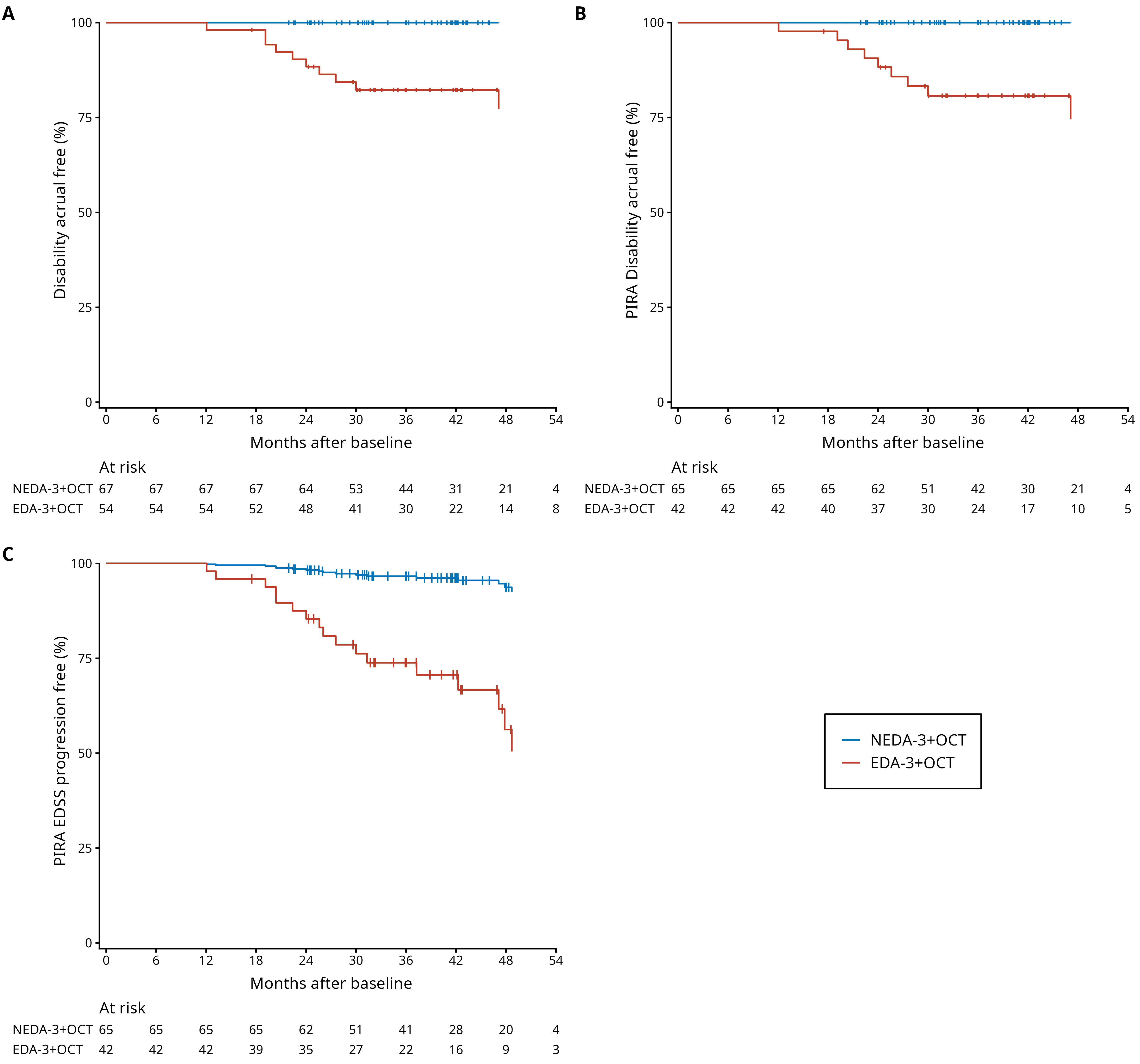
**

**Suppl. Figure S6. Adjusted survival curve according to NEDA-3+OCT status and secondary/tertiary endpoints.** Adjusted survival curves are derived from multivariable Cox proportional-hazards models adjusted for age, sex, disease duration, baseline EDSS, relapse count in the year prior to baseline, baseline MRI T2-lesion count, and baseline DMT category (low- vs. high-efficacy).

NEDA-3/EDA-3 = “No Evidence of Disease Activity – 3”/“Evidence of Disease Activity – 3”; NEDA-3+OCT/EDA-3+OCT = expanded NEDA-3/EDA-3 definition; OCT = Optical coherence tomography; PIRA = Progression independent of relapse activity; EDSS = Expanded disability status scale
