# Supplementary material for: Integration of retinal layer thinning into NEDA-3 predicts disability progression in multiple sclerosis
Source: J Neurol. 2026 Jun 9;273(7):382. doi: 10.1007/s00415-026-13909-8 (PMC13249778; doi:10.1007/s00415-026-13909-8)
Supplement: Supplementary file 2 — Supplementary file2 (DOCX 31 KB) [file 415_2026_13909_MOESM2_ESM.docx]

Supplementary Tables 1-7

|  | Included patients | Whole cohort | p - value |
| --- | --- | --- | --- |
| Age at onset^1^ | 29 (25, 35) | 29 (24, 36) | 0.6^†^ |
| Age at baseline^1^ | 32 (27, 39) | 35 (29, 41) | 0.007^‡^ |
| Number of DMT prior to baseline^1^ | 1 (1, 2) | 1 (1, 2) | 0.7^‡^ |
| Number of relapses prior to baseline^1^ | 1 (1, 1) | 1 (0, 1) | 0.3^‡^ |
| EDSS at baseline^1^ | 2.0 (0, 2.5) | 1.0 (0, 2.0) | 0.038^‡^ |

**Supplementary Table S1. Descriptive comparison of included study population versus whole study cohort (prospective cohort studies).**

^1^Median (IQR)

^†^Welch Two Sample t-test, ^‡^Wilcoxon rank sum test

DMT = Disease-modifying treatment; EDSS = Expanded disability status scale; IQR = Interquartile range; SD = Standard deviation

|  |  |  | 95% CI | |
| --- | --- | --- | --- | --- |
|  | **aHR** | **p-Value** | Lower Limit | Upper Limit |
| EDA-3* | 2.32 | 0.11 | 0.84 | - |
| EDA-3+OCT (pRNFL and/or GCIPL)* | 5.17 | 0.002 | 1.85 | - |
| EDA-3+OCT (pRNFL and/or GCIPL) HE-DMT*† | 5.48 | 0.002 | 1.88 | - |
| EDA-3** | 3.10 | 0.024 | 1.16 | - |
| EDA-3+OCT (pRNFL and/or GCIPL)** | 5.01 | 0.003 | 1.73 | - |
| EDA-3+OCT (pRNFL and/or GCIPL) HE-DMT**† | 5.05 | 0.003 | 1.75 | - |

**Supplementary Table S2. Multivariable Cox Regression analyses for independent variables (EDA-3 or EDA-3+OCT status at one year). Treatment switching was addressed using two complementary approaches: (i) treatment switch modelled as a time-varying covariate, and (ii) censoring at the time of treatment switch before EDSS progression, combined with weighting based on the exposure propensity score and stabilized censoring weights.**

Adjustment of the models with age, sex, EDSS at baseline, disease duration, relapse ≤ 1 year prior to baseline, baseline MRI T2-lesion count and baseline DMT (binary: LE-DMT/HE-DMT) using a propensity-score adjusted approach.

* Treatment switch modelled as a time-varying covariate.

** Censoring at treatment switch before EDSS progression, with combined weighting based on the exposure propensity score and stabilized censoring weights

No multicollinearity was evident (all VIF < 1.7).

A p-value < 0.05 was considered statistically significant, one-sided 95% CI is shown

†Subgroup-specific contrast within the HE-DMT stratum from a Cox-regression model with an interaction term (patients receiving HE-DMT n = 66 [53%])

aHR = Adjusted hazard ratio; CI = Confidence interval; NEDA-3/ EDA-3 = “No Evidence of Disease Activity – 3”/ “Evidence of Disease Activity – 3”; NEDA-3+OCT/ EDA-3+OCT = expanded NEDA-3/EDA-3 definition; OCT = Optical coherence tomography; pRNFL = Peripapillary retinal nerve fiber layer; GCIPL = Ganglion cell and inner plexiform layer; HE-DMT = High efficacy disease-modifying therapy, EDSS = Expanded Disability Status Scale.

|  |  |  |  |  | 95% CI | |
| --- | --- | --- | --- | --- | --- | --- |
|  | **Coefficient** | **SE** | **aHR** | **p-Value** | Lower Limit | Upper Limit |
| EDA-3+OCT (pRNFL and/or GCIPL) | 2.009 | 0.511 | 7.456 | < 0.001 | 2.737 | - |
| EDA-3+OCT (pRNFL) | 1.886 | 0.462 | 6.592 | < 0.001 | 2.507 | - |
| EDA-3+OCT (GCIPL) | 1.855 | 0.478 | 6.392 | 0.001 | 2.531 | - |
| EDA-3+OCT HE-DMT† | 2.194 | 0.847 | 8.968 | 0.01 | 1.706 | - |

**Supplementary Table S3. Multivariable Cox Regression analyses for independent variables (EDA-3+OCT status at one year) using different absolute threshold definitions for OCT progression.** Absolute change from baseline to follow-up OCT was calculated and OCT progression defined as retinal layer thinning of ≥ 2.0 µm/year in pRNFL and/or ≥ 1.0 µm/year in GCIPL. Risk of disability progression grouped according to EDA classification.

Adjustment of the models with age, sex, EDSS at baseline, disease duration, relapse ≤ 1 year prior to baseline, baseline MRI T2-lesion count and baseline DMT (binary: LE-DMT/HE-DMT) using a propensity-score adjusted approach.

No multicollinearity was evident (all VIF < 1.7).

A p-value < 0.05 was considered statistically significant, one-sided 95% CI is shown

†Subgroup-specific contrast within the HE-DMT stratum from a Cox-regression model with an interaction term (patients receiving HE-DMT n = 66 [53%])

aHR = Adjusted hazard ratio; CI = Confidence interval; NEDA-3/ EDA-3 = “No Evidence of Disease Activity – 3”/ “Evidence of Disease Activity – 3”; NEDA-3+OCT/ EDA-3+OCT = expanded NEDA-3/EDA-3 definition; OCT = Optical coherence tomography; pRNFL = Peripapillary retinal nerve fiber layer; GCIPL = Ganglion cell and inner plexiform layer

|  |  |  |  |  | 95% CI | |
| --- | --- | --- | --- | --- | --- | --- |
|  | **Coefficient** | **SE** | **aHR** | **p-Value** | Lower Limit | Upper Limit |
| EDA-3+OCT (pRNFL and/or GCIPL) | 1.497 | 0.519 | 4.467 | 0.005 | 1.585 | - |
| EDA-3+OCT (pRNFL) | 1.368 | 0.462 | 3.929 | 0.002 | 1.838 | - |
| EDA-3+OCT (GCIPL) | 1.461 | 0.478 | 4.312 | 0.001 | 1.964 | - |
| EDA-3+OCT HE-DMT* | 1.871 | 3.950 | 4.75 | 0.031 | 1.21 | - |

**Supplementary Table S4. Multivariable Cox Regression analyses for independent variables (EDA-3+OCT status at one year) using relative threshold definitions for OCT progression.** Relative change from baseline to follow-up OCT was calculated and OCT progression defined as retinal layer thinning of ≥1%/year in pRNFL and/or ≥0.5%/year in GCIPL. Risk of disability progression grouped according to EDA classification.

Adjustment of the models with age, sex, EDSS at baseline, disease duration, relapse ≤ 1 year prior to baseline, baseline MRI T2-lesion count and baseline DMT (binary: LE-DMT/HE-DMT) using a propensity-score adjusted approach.

No multicollinearity was evident (all VIF < 1.7).

A p-value < 0.05 was considered statistically significant, one-sided 95% CI is shown

†Subgroup-specific contrast within the HE-DMT stratum from a Cox-regression model with an interaction term (patients receiving HE-DMT n = 66 [53%])

aHR = Adjusted hazard ratio; CI = Confidence interval; NEDA-3/ EDA-3 = “No Evidence of Disease Activity – 3”/ “Evidence of Disease Activity – 3”; NEDA-3+OCT/ EDA-3+OCT = expanded NEDA-3/EDA-3 definition; OCT = Optical coherence tomography; pRNFL = Peripapillary retinal nerve fiber layer; GCIPL = Ganglion cell and inner plexiform layer; HE-DMT = High efficacy disease-modifying therapy, EDSS = Expanded Disability Status Scale.

|  |  |  |  |  | 95% CI | |
| --- | --- | --- | --- | --- | --- | --- |
|  | **Coefficient** | **SE** | **aHR** | **p-Value** | Lower Limit | Upper Limit |
| EDA-3+OCT (pRNFL/GCIPL)* | 1.365 | 0.561 | 3.914 | 0.015 | 1.304 | - |
| EDA-3+OCT (pRNFL/GCIPL)† | 1.630 | 0.782 | 5.083 | 0.038 | 1.099 | - |
| EDA-3+OCT (pRNFL/GCIPL)‡ | - | - | - | - | - | - |

**Supplementary Table S5. Multivariable Cox proportional-hazards model assessing the association between one-year changes in retinal-layer z-scores and the risk of disability progression (EDSS), stratified according to NEDA classifications**.

Adjustment of the model with age, sex, EDSS at baseline, disease duration, relapse ≤ 1 year prior to baseline, baseline MRI T2-lesion count and baseline DMT (binary: LE-DMT/HE-DMT) using a propensity-score adjusted approach.

Receiver-operating-characteristic (ROC) analyses yielded optimal absolute *z*-score difference thresholds of 0.11 for pRNFL and 0.064 for GCIPL to discriminate NEDA-3+/EDA-3+OCT status.

No multicollinearity was evident (all VIF < 1.7).

Statistical significance was set at p < 0.05; one-sided 95 % confidence intervals are reported.

* Primary endpoint

† PIRA-EDSS

‡ Secondary endpoint (No events in NEDA+3-OCT group. Complete separation precluded estimation of a HR, and the Cox model failed to converge [infinite coefficient].)

aHR = Adjusted hazard ratio; CI = Confidence interval; NEDA-3/EDA-3 = “No Evidence of Disease Activity – 3”/“Evidence of Disease Activity – 3”; OCT = Optical coherence tomography; NEDA-3+OCT/EDA-3+OCT = expanded NEDA-3/EDA-3 definition; pRNFL = Peripapillary retinal nerve fiber layer; GCIPL = Ganglion cell and inner plexiform layer; HE-DMT = High efficacy disease-modifying therapy, EDSS = Expanded Disability Status Scale

|  |  |  |  |  | 95% CI | |
| --- | --- | --- | --- | --- | --- | --- |
|  | **Coefficient** | **SE** | **aHR** | **p-Value** | Lower Limit | Upper Limit |
| EDA-3+OCT (pRNFL, lower third) | 3.516 | 1.213 | 33.6646 | 0.004 | 3.125 | - |
| EDA-3+OCT (pRNFL, upper third) | 2.048 | 3.988 | 7.806 | 0.999 | NC | - |
| EDA-3+OCT (GCIPL, lower third) | 2.827 | 1.131 | 16.902 | 0.013 | 1.84 | - |
| EDA-3+OCT (GCIPL, upper third) | 0.856 | 0.855 | 2.355 | 0.317 | 0.441 | - |

**Supplementary Table S6 Multivariable Cox proportional-hazards model assessing the layer-specific association between EDA-3+OCT status and the primary endpoint grouped by baseline thickness.**

Adjustment of the model with age, sex, EDSS at baseline, disease duration, relapse ≤ 1 year prior to baseline, baseline MRI T2-lesion count and baseline DMT (binary: LE-DMT/HE-DMT) using a propensity-score adjusted approach.

No multicollinearity was evident (all VIF < 1.7).

Statistical significance was set at p < 0.05; one-sided 95 % confidence intervals are reported.

SE = Standard error; aHR = Adjusted hazard ratio; CI = Confidence interval; NEDA-3/EDA-3 = “No Evidence of Disease Activity – 3”/“Evidence of Disease Activity – 3”; OCT = Optical coherence tomography; NEDA-3+OCT/EDA-3+OCT = expanded NEDA-3/EDA-3 definition; EDSS = Expanded disability status scale; pRNFL = Peripapillary retinal nerve fiber layer; GCIPL = Ganglion cell and inner plexiform layer; NC = Not computable

|  |  |  |  |  | 95% CI | |
| --- | --- | --- | --- | --- | --- | --- |
|  | **Coefficient** | **SE** | **aHR** | **p-Value** | Lower Limit | Upper Limit |
| PIRA-EDSS | 2.179 | 0.703 | 8.842 | 0.002 | 2.23 | - |
| EDSS progression with disability accrual* | - | - | - | - | - | - |
| PIRA-EDSS with disability accrual* | - | - | - | - | - | - |

**Supplementary Table S7. Multivariable Cox proportional-hazards model assessing the association between EDA-3+OCT status and secondary and tertiary endpoints.**

Adjustment of the model with age, sex, EDSS at baseline, disease duration, relapse ≤ 1 year prior to baseline, baseline MRI T2-lesion count and baseline DMT (binary: LE-DMT/HE-DMT) using a propensity-score adjusted approach.

*Complete separation precluded estimation of a HR, and the Cox model failed to converge (infinite coefficient)

No multicollinearity was evident (all VIF < 1.7).

Statistical significance was set at p < 0.05; one-sided 95 % confidence intervals are reported.

aHR = Adjusted hazard ratio; CI = Confidence interval; NEDA-3/EDA-3 = “No Evidence of Disease Activity – 3”/“Evidence of Disease Activity – 3”; OCT = Optical coherence tomography; NEDA-3+OCT/EDA-3+OCT = expanded NEDA-3/EDA-3 definition; EDSS = Expanded disability status scale; PIRA-EDSS = Progression independent of relapse activity; MRI = Magnetic resonance imaging; LE-DMT = Low-efficacy disease modifying treatment; HE-DMT = High-efficacy disease modifying treatment
